# Apolipoprotein E ε4: A Possible Risk Factor of Intracranial Pressure and White Matter Perfusion in Good-Grade Aneurysmal Subarachnoid Hemorrhage Patients at Early Stage

**DOI:** 10.3389/fneur.2017.00150

**Published:** 2017-04-19

**Authors:** Jian-hua Peng, Xing-hu Qin, Jin-wei Pang, Yue Wu, Jin-hu Dong, Chang-ren Huang, Wei-feng Wan, Xiao-bo Yang, Xiao-chuan Sun, Li-gang Chen, Yong Jiang

**Affiliations:** ^1^Department of Neurosurgery, The Affiliated Hospital of Southwest Medical University, Luzhou, China; ^2^Department of Neurosurgery, People’s Hospital of Deyang City, Deyang, China; ^3^Department of Neurosurgery, The First Affiliated Hospital of Chongqing Medical University, Chongqing, China

**Keywords:** apolipoprotein E, subarachnoid hemorrhage, early brain injury, intracranial pressure, computed tomography perfusion, white matter injury

## Abstract

Aneurysmal subarachnoid hemorrhage (aSAH) is a devastating and complicated disease with significant morbidity and mortality. Previous studies have shown that genetic susceptibility may play an important role in the outcome of a given individual with aSAH. This study evaluates the potential association in effects of the APOE allele on the early brain injury (EBI) in light of elevated intracranial pressure (ICP) and cerebral perfusion disorders in a consecutive series of non-comatose Chinese patients with aSAH. A total of 122 patients with aSAH (54 males and 68 females) were enrolled in this study. Demographic and clinical data were collected. We measured ICP before microsurgical clipping or endovascular coiling during the first 72 h after aneurysm rupture. Computed tomography perfusion (CTP) examination in patients was performed before treatment. The distributions of APOE genotypes and alleles matched Hardy–Weinberg law (*p* > 0.05). In this study, 68 patients (55.7%) had a normal ICP, whereas 54 (44.3%) had an elevated ICP. Fourteen of 21 patients with APOE ε4 had an elevated ICP, which was significantly different from those without APOE ε4 (*p* = 0.03). The patients with the ε4 allele had a higher incidence of elevated ICP [*p* = 0.009, 95% confidence interval (CI) = 1.481–15.432, odds ratio = 4.780] than those without this allele. For CTP measurements, a lower mean cerebral blood flow (difference, −4.74; 95% CI, 0.53–8.94 s, *p* = 0.03), longer mean transit time (difference, 0.47; 95% CI, −0.87 to −0.78, *p* = 0.02), and time-to-peak (difference, 2.29; 95% CI, −3.64 to −0.93 s, *p* = 0.02) were observed in patients with ε4 allele than in those without in the internal capsule regions. In conclusion, the APOE ε4 allele predisposes patients to elevated ICP and perfusion disorders in white matter regions during the first 72 h after aSAH. The presence of an APOE ε4 allele plays an important role in the EBI response to aSAH.

## Introduction

Aneurysmal subarachnoid hemorrhage (aSAH) is a devastating and complicated disease with significant morbidity and mortality (1). Traditionally, cerebral vasospasm (CVS) and aneurismal rebleeding have been considered to be major complications that significantly worsen the prognosis of aSAH (2). In recent years, early brain injury (EBI) during the first 72 h after aSAH has been shown to play an important determinant of clinical outcome (3). Notably, global cerebral ischemia as a hallmark of EBI is thought to be related to raised intracranial pressure (ICP) and cerebral perfusion (4–6). Cerebral perfusion is related to mean arterial blood pressure and ICP, which reduces the cerebral perfusion and threatens the brain function.

Increasing evidence has shown that aSAHs with similar pathologies might have different clinical statuses and outcomes. However, the Hunt and Hess grade, sex, and age of the patient can explain only part of this variability. Alternatively, genetic susceptibility may play an important role in the outcome of a given individual with aSAH (7, 8). Recently, apolipoprotein E (APOE = gene; apoE = protein) has become one of the most widely studied genes in aSAH research (9). There are three common isoforms (apoE2, apoE3, and apoE4) encoded by three gene alleles (APOE ε2, APOE ε3, and APOE ε4) (10). Although the presence of the APOE ε4 allele predisposes poorer outcome in patients with aSAH, the conclusions of investigations are still conflicting (11, 12).

Increased ICP is common after SAH, even in patients with a good clinical H–H grade. Elevated ICP post-SAH is associated with a worse patient outcome (13). Patients with good-grade aSAH (Hunt and Hess grades I–III) can also die or present severe deficit due to increased ICP leading to cerebral ischemia (14). Elevated ICP post-SAH is associated with good-grade patient outcomes (13). Our previous study indicated that APOEε4 as an independent risk factor for cerebral perfusion dysfunction (6). However, the risk of APOE genotype on ICP and cerebral perfusion of good-grade aSAH patients has never been investigated in detail. The present study aimed to evaluate the potential association between APOE allele and the progression of EBI in light of increased ICP and cerebral perfusion in a consecutive series of good-grade Chinese patients with aSAH.

## Materials and Methods

### Study Design and Inclusion and Exclusion Criteria

A total of 122 consecutive patients with onset of aSAH shorter than 72 h were enrolled to the two departments of neurosurgery in this prospective pilot study from December 2014 to July 2016 (The Affiliated Hospital of Southwest Medical University: 67 patients and The First Affiliated Hospital of Chongqing Medical University: 55 patients). aSAH was confirmed using CT scans and lumbar puncture. The sample group of adult patients included 54 males and 68 females, aged 48–72 years old, with a diagnosis of SAH from a ruptured cerebral aneurysm, which was verified using digital subtraction angiography or CT angiography.

The following inclusion criteria were applied: (i) the clinical grade before treatment was I–III, according to the Hunt and Hess grading system (e.g., good-grade non-comatose patients); (ii) all of the patients were treated within 72 h with early microsurgical clipping; and (iii) clinical data were recorded completely.

We excluded patients with the following characteristics: (i) cerebral lesions other than aneurysms because they can be independent predictors of ICP increases and can render the attribution of ICP less reliable; (ii) displayed symptomatic CVS with a PMV ≥ 120 cm/s (15) or severe narrowing of the involved artery on cerebral angiogram within the first 72 h; and (iii) aneurysmal rebleeding, intra-ventricular hemorrhage, or hydrocephalus observed by CT scan within 72 h.

### Recording of Clinical Information

The clinical data of the included patients were recorded; this information included the patient’s age, sex, neurological condition before treatment (Hunt–Hess grade), and Fisher grade on admission as well as the site of the ruptured aneurysm, and the type of treatment (clipping or coiling).

### APOE Genotype

APOE genotype was determined from genomic DNA extracted from venous blood that was collected from the patients upon admission. DNA was extracted from frozen blood using standard techniques, and APOE ε2, ε3, and ε4 genotyping was performed using the polymerase chain reaction–restriction fragment length polymorphism method, as previously described (16). Briefly, a 250-bp fragment containing the coding region was amplified. The following primers were used: P1: 5′-TAAGCTTGGCACGGCTGTCCAAGGA-3′ (upstream) and P2: 5′-ACAGAATTCGCCCCGGCCTGGTACAC-3′ (downstream). The PCR products were digested using the *Hha*I restriction enzyme, and the fragments were separated by electrophoresis on 4% ethidium bromide-containing agarose gels for genotype determination.

### ICP Monitoring

Lumbar puncture allowed us to gage the ICP of the SAH patients (17). Thus, lumbar pressure (LP) was used in this study as a surrogate measurement of ICP. After the source of SAH was identified, either aneurysm clipping or coiling was performed within 72 h. Before the operation, a silicon lumbar catheter was introduced through a 16-G lumbar needle into the subarachnoid space at the L4–L5 level. This catheter was connected to a sterile collecting system fixed above the point of catheter insertion. The LP was measured *via* a hydrostatic pressure transducer, which was immediately connected to the drainage system. The zero reference pressure was the atmospheric pressure at the level of the foramen of Monro. The ICP threshold was set at 20 mmHg because this pressure is the recommended threshold for routine initiation of ICP treatment in neurosurgery in adults.

### Computed Tomography Perfusion (CTP) Screening

To characterize the region-specific response to cerebral perfusion, CTP examination in patients was performed before treatment. After injection 30 ml of non-ionic contrast agent (iopromide Ultravist, 370 mg iodine/ml; Schering, Berlin, Germany) and 15 ml of normal saline through the cubital vein (4 ml/s), head scanning was performed by a Lightspeed VCT 64-slice CT machine. The following parameters were used: 80 kVp, 200 mAs, 5 mm slice thickness. Original data were transferred to an Adw4.2 workstation for subsequent analysis. The measurements of arterial input (anterior cerebral artery) and venous output (superior sagittal sinus) functions were semiautomatic. We cooperated with the clinical radiologists because they will likely become expert in distinguishing CT images on a region-by-region basis. Hand-drawn, multi regions of interest (ROIs) were defined by prespecified anatomical boundaries on CT images. One side and the contralateral mirror area of caudate nucleus, internal capsule, thalamus, external capsule, and whole cortex were scanned as the ROI. The cerebral blood volume, cerebral blood flow (CBF), mean transit time (MTT), and time-to-peak (TTP) were obtained. To ensure the accuration of each ROI, we narrowed ROIs in regions with indistinguishable boundary, and all the ROIs of each patient were drawn by the same assessor.

### Statistical Analysis

SPSS software (version 19.0) was used for statistical analyses. After counting the number of alleles, the allele frequencies in aSAH patients were calculated from the sample proportions. The constituent ratio of each correlation factor in the clinical data between the groups (with ε4 allele and without ε4 allele) was analyzed using the χ^2^ test for nominal variables. Univariate logistic regression was performed to analyze the associations of age (≤65 years old vs. >65 years old), Hunt–Hess grade (1 and 2 vs. 3), Fisher grade (1 and 2 vs. 3 and 4), aneurysm site (anterior communicating artery vs. other sites), type of treatment (clipping vs. coiling), and APOE genotype (with ε4 vs. without ε4) with elevated ICP. After adjusting the clinical data, further multivariate logistic regression analyses (stepwise backward conditional and Wald functions, as appropriate) were used to analyze the associations of the APOE genotype (with ε4 vs. without ε4) with elevated ICP. Variables entered into the multivariate regression at *p* < 0.05, while some variables were removed at *p* > 0.1. Adjusted odds ratios (ORs) and 95% confidence intervals (CIs) were calculated from the logistic regression model coefficients. In addition, we compared differences in mean values of cerebral perfusion between patients with and without ε4 allele by calculating the 95% CIs of the mean difference. The statistical significance of the correlation between the investigated quantitative variables was determined by the *p* value, and a *p* value <0.05 was considered significant.

## Results

The department admitted 210 patients with aSAH during the study period. Seventy-one patients were not included in the study because their clinical conditions were classified as IV or V on the Hunt and Hess grading scale. Seventeen good-grade patients met the exclusion criteria and were disqualified: two patients had cerebral lesions other than aneurysms, six patients had aneurysmal rebleeding or hydrocephalus within 72 h, three patients had intracerebral hematomas, and six patients displayed symptomatic CVS, with a PMV ≥ 120 cm/s, or suffered from severe narrowing of the involved artery as observed on cerebral angiography within 72 h. The remaining 122 aSAH patients were enrolled in the current study.

Of the 122 patients, allele frequencies were 9.4% for the ε4 allele and 90.6% for the non-ε4 allele. The distributions of the APOE allele frequencies and genotypes are presented in Table 1. The samples demonstrated Hardy–Weinberg equilibrium (*p* > 0.05).

**Table 1:** **Distribution of APOE genotype**.

Genotype						Allele frequency		
ε2/2	ε2/3	ε2/4	ε3/3	ε3/4	ε4/4	ε2	ε3	ε4
0	15	4	86	15	2	7.8%	82.8%	9.4%

The clinical data of the included patients are summarized in Table 2; these data include age, sex, Hunt and Hess grade and Fisher grade of CT scans, aneurysm site, and the type of treatment. The distribution of the baseline characteristics between the groups of patients with and without an APOE ε4 allele was not different (*p* > 0.05).

**Table 2:** **Baseline characteristics of patients with and without ε4 allele**.

	Total series (*n* = 122)	Patients with ε4 allele (*n* = 21)	Patients without ε4 allele (*n* = 101)
**Age**			
≤65 years	80	10	70
>65 years	42	11	31
**Sex**			
Male	54	9	45
Female	68	12	56
**H–H grade**			
1 and 2	64	11	53
3	58	10	48
**Fisher grade**			
1 and 2	53	13	40
3	69	8	61
**Site**			
AcoA	50	9	41
Other	72	12	60
**Treatment**			
Clipping	98	16	81
Coiling	24	5	19

### Effect of APOE ε4 Allele on ICP

All of the ICP analyses were based on values obtained from lumbar drainage. The patients were separated into two groups based on their LP values before treatment. ICP values of 20 mmHg were chosen because an ICP greater than 20 mmHg is a well-known threshold for treating patients with neurosurgery. Therefore, the normal ICP group included patients who had an ICP less than 20 mmHg, and the elevated ICP group consisted of patients with an ICP greater than 20 mmHg. In this study, 68 patients (55.7%) had a normal ICP, whereas 54 (44.3%) had an elevated ICP. The ICP was increased in good-grade patients with a Hunt and Hess grade of I–III. Fourteen of 21 patients with APOE ε4 had an elevated ICP, which was significantly different from those without APOE ε4 (*p* = 0.03). According to the univariate analysis, patients with the ε4 allele had a higher incidence of elevated ICP than those without this allele (*p* = 0.03, 95% CI = 1.132–8.217, OR = 3.1). When the data were adjusted for significant risk factors in the multivariate analysis (age, sex, Hunt and Hess grade, Fisher grade, etc.), the association of the ε4 allele with the risk of elevated ICP was even more significant (*p* = 0.009, 95% CI = 1.481–15.432, OR = 4.780). Furthermore, elevated ICP was also associated with a worse Hunt–Hess grade (*p* = 0.004, 95% CI = 1.536–9.116, OR = 3.742) (Table 3). The patient’s age, sex, Fisher grade, and aneurysm site were not statistically associated with ICP.

**Table 3:** **Intracranial pressure logistic regression**.

Characteristic	Univariate			Multivariate		
	*p* Value	OR	95% CI	*p* Value	OR	95% CI
APOEε4	0.027*	3.1	1.132–8.217	0.009*	4.780	1.481–15.432
Sex	0.256	0.7	0.320–1.355	0.438	0.717	0.309–1.664
Age	0.821	0.9	0.432–1.947	0.416	0.669	0.254–1.762
H–H grade	0.022*	2.4	1.132–4.879	0.004*	3.742	1.536–9.116
Fisher grade	0.866	1.1	0.517–2.189	0.133	2.013	0.809–5.008
Site	0.247	1.5	0.740–3.218	0.022	3.013	1.176–7.717

*OR, odds ratio; CI, confidence interval*.

*Significant difference (*p* < 0.05).

### Assessment of the APOE ε4 Allele and Cerebral Perfusion

**Figure 1:**
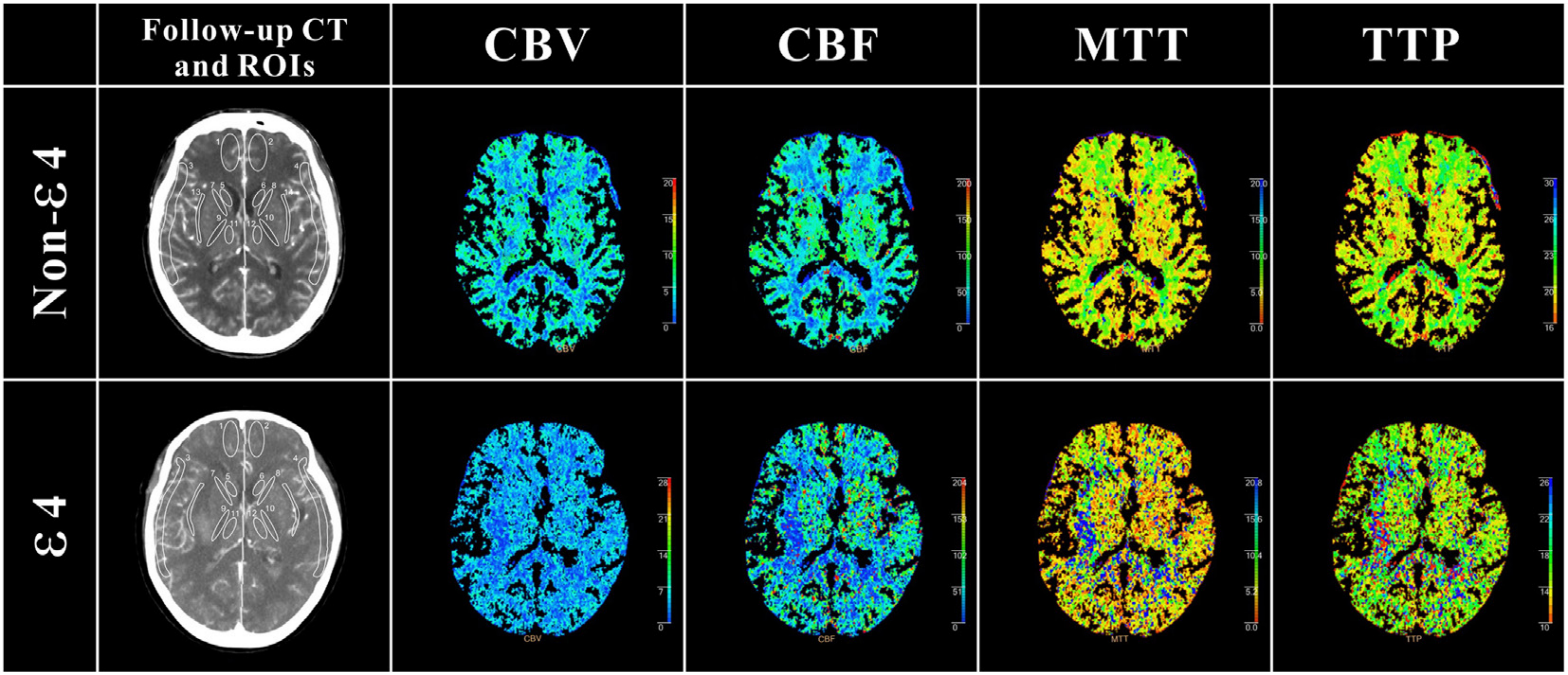
**Definition of regions of interest (ROIs) in follow-up CT and cerebral perfusion images in ε4 and non-ε4 SAH patients**. ROIs were drawn in 5 different regions: cortex: ROIs 1 and 2 (anterior cerebral artery), ROIs 3 and 4 (middle cerebral artery); caudate nucleus: ROIs 5 and 6; internal capsule: ROIs 7 and 8 (anterior limb), ROIs 9 and 10 (posterior limb); thalamus external: ROIs 11 and 12; capsule: ROIs 13 and 14. As shown in cerebral perfusion images, APOE ε4 allele aneurysmal subarachnoid hemorrhage patients suffered more severe perfusion disorders, especially in white matter regions.

As shown in Table 4, in the internal capsule area, CBF was 33.03 mL/100 g/min in patients with ε4 allele and 37.77 mL/100 g/min in those without ε4 allele (difference, −4.74; 95% CI, 0.53–8.94 s, *p* = 0.03). In patients with APOE ε4, MTT was 6.29 s, and in those without ε4 allele, MTT was 5.33 s in internal capsule (difference, 0.47; 95% CI, −0.87 to −0.78 s, *p* = 0.02). As was TTP, patients with ε4 allele had a significantly longer TTP than patients without ε4 allele both in internal capsule (difference, 2.29; 95% CI, −3.64 to −0.93 s, *p* = 0.02) and external capsule (difference, 1.09; 95% CI, −2.16 to −0.01 s, *p* = 0.04). These results suggested that patients with ε4 allele had a significant abnormality in brain cerebral perfusion in internal capsule and other whiter matter regions, such as external capsule (Figure 1).

**Table 4:** **Cerebral perfusion in patients with and without ε4 allele**.

APOEε4	Mean CBV (mL/100 g)		Mean CBF (mL/100 g/min)		Mean MTT (s)		Mean TTP (s)	
	Yes	No	Yes	No	Yes	No	Yes	No
Caudate nucleus	4.28 ± 2.36	4.19 ± 2.58	52.01 ± 6.56	54.45 ± 7.78	4.58 ± 2.93	4.17 ± 1.7	24.36 ± 1.52	23.43 ± 1.28
Difference of means (95% CI; *p* value)	0.09 (−1.79 to 1.61; *p* = 0.91)		−2.44 (−2.59 to 7.46; *p* = 0.33)		0.41 (−2.08 to 1.26; *p* = 0.62)		0.93 (−1.91 to −0.52; *p* = 0.06)	

Internal capsule	3.07 ± 0.86	3.15 ± 1.29	33.03 ± 5.50	37.77 ± 6.49	6.29 ± 0.71	5.82 ± 0.25	28.43 ± 1.89	26.14 ± 1.98
Difference of means (95% CI; *p* value)	0.08 (−0.69 to 0.85; *p* = 0.83)		−4.74 (0.53 to 8.94; *p = 0.03)		0.47 (−0.87 to −0.78; *p = 0.02)		2.29 (−3.64 to −0.93; *p = 0.02)	

Thalamus	3.78 ± 1.14	3.62 ± 1.04	41.98 ± 6.93	43.95 ± 6.68	5.36 ± 0.91	5.04 ± 1.01	21.87 ± 1.81	22.11 ± 2.26
Difference of means (95% CI; *p* value)	0.16 (−0.93 to 0.61; *p* = 0.67)		−4.04 (−2.78 to 6.72; *p* = 0.40)		0.32 (−0.98 to 0.35; *p* = 0.35)		−0.24 (−1.18 to 1.67; *p* = 0.73)	

External capsule	3.34 ± 0.86	3.20 ± 1.05	36.21 ± 7.21	39.96 ± 7.64	6.24 ± 1.41	5.99 ± 1.30	25.69 ± 1.46	24.60 ± 1.61
Difference of means (95% CI; *p* value)	0.14 (−0.81 to 0.53; *p* = 0.67)		−3.75 (−1.44 to 8.94; *p* = 0.15)		0.26 (−1.2 to 0.69; *p* = 0.59)		1.09 (−2.16 to −0.01; *p = 0.04)	

Cortex	4.17 ± 1.13	4.00 ± 1.04	50.71 ± 8.40	52.31 ± 7.32	4.91 ± 1.21	4.83 ± 1.39	24.08 ± 2.61	22.40 ± 2.66
Difference of means (95% CI; *p* value)	0.17 (−0.93 to 0.57; *p* = 0.63)		−1.60 (−3.90 to 7.10; *p* = 0.15)		0.08 (−0.99 to 0.82; *p* = 0.86)		1.68 (−3.53 to 0.16; *p* = 0.07)	

*CBV, cerebral blood volume; CBF, cerebral blood flow; MTT, mean transit time; TTP, time-to-peak; CI, confidence interval*.

*Significant difference (*p* < 0.05).

## Discussion

From the study of 122 good-grade aSAH patients, our major finding is that those aSAH patients carrying the APOE ε4 allele are predisposed to elevate ICP within 72 h of the ictus, after controlling for age, the severity of the hemorrhage, the aneurysm location, and other factors. Moreover, patients with ε4 allele had a significant abnormality in brain cerebral perfusion in white matter (especially in the internal capsule area). The current study provides some initial clinical evidence that the APOE ε4 allele is associated with a detrimental effect on a patient’s early response to aSAH.

Recently, particular attention has been paid to the APOE gene. apoE is associated with complex neuroprotective functions in the biochemical network of SAH (10, 18, 19). However, the neuroprotective effectiveness of the apoE4 isoform is reduced when compared with the other isoforms, and this decreased effectiveness has designated the ε4 allele as a sort of “frailty protein” and a potential risk factor. Several possible mechanisms might be involved in different ways (10, 18): (i) apoE4 might be a defective free radical scavenger; (ii) patients with the apoE4 protein could have impaired modulation of the brain inflammatory response to SAH, due to unregulated cytokine cascades; or (iii) reduced membrane repair and synaptic plasticity might occur.

Although controversial, increasing evidence indicates that the APOE ε4 genotype predisposes the patient to a poor outcome following aSAH (11, 12). In the Chinese population, the ε4 allele is also thought to be a possible risk factor for poor outcomes following aSAH (20). In our previous work, a series of follow-up clinical studies also demonstrated that patients with the APOE ε4 allele were prone to CVS and brain dysfunction, instead of rebleeding, after spontaneous SAH, which can also contribute to poor outcomes (21, 22).

In aSAH patients, global hypoperfusion caused by increased ICP and focal ischemia induced by vasospasm are common and devastating outcomes. In recent decades, CVS has become the focus of a large number of clinical and experimental research efforts. Although vasospasm is regarded as the primary cause of mortality and neurological morbidity in patients initially surviving aSAH, therapies designed to prevent and treat vasospasm remain limited (4, 23). Increasing evidence has led researchers to cast doubt on the importance of vasospasm in the setting of SAH. In recent years, EBI has been suggested to play a key role in aSAH patients’ clinical deterioration and subsequent poor outcomes.

A previous study conducted on non-comatose patients with aSAH found that the presence of the ε4 allele increased the risk of clinical vasospasm, enhanced cognitive morbidity, and delayed ischemic neurologic deficit recovery (24). As increased ICP is common after aSAH, even in patients with good clinical grades, outcomes are negatively correlated with increased ICP. ICP appears to be a stronger predictor than the influence of vasospasm (25). To our knowledge, this relationship has not been reported in the clinical studies exploring the APOE gene’s influence on ICP in the early stages of aSAH. In the present study, we measured ICP using lumbar drainage before surgery as a surrogate measurement, and we demonstrated that patients with the APOE ε4 polymorphism were predisposed to the presence of elevated ICP during the early phases of aSAH. In one TBI study, APOEε4 could increase cellular and tissular vulnerability (26), Olivecrona and coworkers found that there was no statistically significant difference in the ICP or cerebral perfusion pressure (CPP) of ε4 and non-ε4 patients with severe TBI who were treated with an ICP-targeted therapy (24). These results are apparently in contrast with those of our study. However, the two reports are not actually comparable because of their different neurological diseases, inclusion/exclusion criteria, and so on.

Early brain injury is believed to arise from significant pathophysiological mechanisms that occur in the brain during aSAH (2, 3). The acute rise in ICP, which has been demonstrated in both clinical studies and experimental animal models, results from the initial mass effect of the blood flowing into the subarachnoid space (27, 28), impeded CSF drainage (29), cerebrovascular dysfunction (30), and the development of cerebral edema (31). Elevated ICP may be correlated with the extent of EBI (32). As the pressure rises, a compensatory decrease in CPP occurs. The mechanism behind this relationship may be related to the Monroe–Kelly hypothesis. If the compensatory mechanism is not sufficient to support critical levels of brain perfusion, increased ICP and subsequently decreased CPP can result in significant reductions in CBF, leading to the deterioration of neurological function. apoE4 exerts a strong influence of cerebrovasculature dysfunction and disrupts CBF (33). In our previous study, APOEε4 carriers exhibit MTT prolongation in CTP scanning within 24 h (6). We further measured the region-specific response to cerebral perfusion in this study. We found a significant prolongation of MTT and TTP in the internal capsule and TTP in the external capsule of patients with ε4 allele. Although the reduction of values are of small differences, the ε4 aSAH patients presented a lower CBF in regions measured in this study, while the internal capsule region presented a significant difference. Furthermore, the MTT prolonged over 5.9 s in some regions, such as internal capsule. Our previous study indicated APOEε4 as an independent risk factor for prolonged MTT over 5.9 s, suggestive of increased risk of DCI and poor outcome in APOEε4 patients (6). Meanwhile, a significant reduction of CBF was seen in internal capsule of patients with ε4 allele. Previous study demonstrates that astrocytes are capable of eliciting both vasoconstriction and vasodilation, then control of CBF in brain (34). The apoE4-induced detrimental changes may be linked to astrocyte activation (33). Although several limitations of CTP should be considered, this technology may reflect the white matter injury indirectly according to regional perfusion. The abnormality in brain cerebral perfusion in white matter regions suggested that patients with ε4 allele might suffer more serious white matter injury after aSAH.

White matter injury has recently been reported in clinical and experimental SAH (35, 36). Victims of SAH suffer most from global emotional and cognitive dysfunction and associated diffuse cerebral atrophy, and diffuse axonal injury may also be an important feature of SAH (37, 38). van Asch and coworkers also observed an abnormalities perfusion of white matter in acute hydrocephalus aSAH patients (39). Studies support the hypothesis that white matter injury is due to CVSs and perfusion disorders after a brain injury (40–42). Although this has been identified as the only proven drug to improve patients’ outcomes after SAH, nimodipine, a kind of calcium antagonist, needed to advance the understanding of its effects on microcirculatory changes and cerebral perfusion after SAH. Thus, development of feasible SAH therapeutic strategies still needs more effort. Our previous study has identified ApoE-mimetic peptide COG1410 in EBI by preventing white matter injury after experimental SAH (43). Because ApoE-mimetic peptide is a modified peptide sequence from human apoE, this could indicate that apoE4 lacks a protective effect found in apoE2 and apoE3 in the biochemical network of SAH. Hence, APOEε4 may induce cerebral perfusion impairment *via* elevated ICP in the early phase, contributing to white matter injury in EBI following aSAH. Furthermore, increased postoperative ICP is common after early clipping, especially in poor-grade aSAH patients, and this might precipitate CVS-induced ischemic events in aSAH patients including the deterioration of neurological function and poor outcomes (44, 45). Patients with symptomatic CVS were excluded in this study, the results indicated that one of the most important factors in predicting the outcomes is correlated with elevated ICP, including good H–H grade aSAH patients with ε4 allele.

Although no previous clinical studies have specifically focused their attention on ICP after SAH, these results are supported by the experimental observations that wild-type mice treated with ApoE-mimetic peptide after experimental SAH decreased mortality, functional deficits and vasospasm (25), neurons apoptosis (46), and BBB disruption (47) as compared with vehicle-treated mice. Notably, in that study, SAH-affected mice expressing the human ε4 allele were prone to higher mortality rates and greater functional deficits compared to their human ε3 counterparts during the first 72 h after experimental SAH. These deficits were associated with a greater degree of cerebral edema and vasospasm in mice expressing the apoE4 isoform. Modulation of the CNS inflammatory response might be one mechanism by which APOE genotype affects EBI after SAH.

### Limitations

There were several potential limitations of the current study. First, as blood is mainly located in the basal cisterns in aSAH patients, CSF outflow from the ventricles into the subarachnoid space should not be substantially impaired. At the same time, the location of the blood clot can present a barrier to the downstream flow of CSF from the convex subarachnoid space of the cerebral hemispheres through the foramen magnum into the spinal compartment. Thus, a partial obstruction might be present and render the LP lower than the actual ICP. Second, although lumbar drainage has been shown to be safe in SAH patients, the formation of pressure gradients remains a potential threat that could result in herniation and rebleeding. Therefore, lumbar drainage was only performed in the present study before aneurysm clipping or coiling. An additional method, such as continuous ICP monitoring, might be needed to verify our findings. Third, abnormality in brain cerebral white matter perfusion of ε4 allele aSAH patients was observed in this study. However, the specific reason of white matter injury after SAH is not entirely clear. The detail pathophysiological processes and clinical diagnosis strategy of white matter injury after SAH need to be further investigated both in experimental and clinical studies.

### Conclusion and Outlook

We evaluated the association between the presence of the APOE ε4 allele and elevated ICP *via* invasive (LP) in this prospective study. Despite the limitations of the present study, these preliminary findings provide new evidence that the APOE ε4 allele predisposes patients to elevated ICP after aSAH. Meanwhile, APOE ε4 allele aSAH patients suffered more severe perfusion disorders in white matter regions. These clinical observations support the hypothesis that APOE plays an important role in the response of the CNS to EBI, possibly involving the adverse effects of apoE4 on white matter injury, neurobiology, and its potential links to certain risk factors, such as elevated ICP. To improve the precision medicine, further studies are needed to elucidate the mechanisms by which the APOE ε4 allele can adversely affect a patient’s response to EBI after aSAH.

## Ethics Statement

The study was approved by the ethics committee of the coordinating institution and has therefore been performed in accordance with the ethical standards laid down in the 1964 Declaration of Helsinki and its later amendments. All the patients gave their informed consent prior to their inclusion in the study. Informed consent was obtained from each patient directly.

## Author Contributions

Conception and design: YJ and J-hP. Acquisition of data: J-wP, X-hQ, and X-bY. Analysis and interpretation of data: YW, J-hD, W-fW, and C-rH. Drafting the article: J-hP and YJ. Reviewed submitted version of manuscript: YJ, X-cS, and L-gC. Statistical analysis: YW, J-hD, and J-wP. Study supervision: YJ, X-cS, and L-gC.

## Conflict of Interest Statement

The authors declare that the research was conducted in the absence of any commercial or financial relationships that could be construed as a potential conflict of interest.
